# The Repertoire of Adenovirus in Human Disease: The Innocuous to the Deadly

**DOI:** 10.3390/biomedicines6010030

**Published:** 2018-03-07

**Authors:** Subrat Khanal, Pranita Ghimire, Amit S. Dhamoon

**Affiliations:** Department of Medicine, State University of New York, Upstate Medical University, Syracuse, NY 13210, USA; ghimirep@upstate.edu (P.G.); dhamoona@upstate.edu (A.S.D.)

**Keywords:** adenovirus, biology, immunocompetent, immunocompromised

## Abstract

Adenoviridae is a family of double-stranded DNA viruses that are a significant cause of upper respiratory tract infections in children and adults. Less commonly, the adenovirus family can cause a variety of gastrointestinal, ophthalmologic, genitourinary, and neurologic diseases. Most adenovirus infections are self-limited in the immunocompetent host and are treated with supportive measures. Fatal infections can occur in immunocompromised patients and less frequently in the healthy. Adenoviral vectors are being studied for novel biomedical applications including gene therapy and immunization. In this review we will focus on the spectrum of adenoviral infections in humans.

## 1. Introduction

Adenoviruses are ubiquitous, double-stranded DNA viruses that are most commonly associated with pediatric illnesses of the upper respiratory tract, including the common cold. Adenoviral infections can also manifest with gastrointestinal, ophthalmologic, genitourinary, and neurologic symptoms. In this discussion we will review the pathobiology of the virus and the broad clinical spectrum of disease manifestation in immunocompetent and immunocompromised patients. We will review global trends along with management strategies.

## 2. Structure

The adenoviridae family is comprised of relatively large, icosahedral, non-enveloped viruses with linear, double-stranded DNA [1], classified into seven species (A to G) [2]. Structurally, adenovirus is composed of two major elements, the outer capsid and the inner core, in which viral dsDNA genome is enclosed together with a large number of histone-like proteins. Viral proteases play a major role in the maturation by cleavage of precursor proteins for the capsid and the core. Over 60 human adenovirus [2] serotypes have been identified based on antigenic determinants detected by viral neutralization assays and hemagglutination studies.

## 3. Biology of Infection

Adenoviral infection is initiated by elongated fiber proteins from the virus interacting with multiple host cell receptors resulting in cell attachment and internalization. Two of the most-studied host receptors are the CAR (coxsackie and adenovirus receptor [3]) found on multiple polarized epithelial cells and the MCP (membrane cofactor protein [4]) or CD46, widely distributed on the surface of many different host cells. For most adenovirus groups, CAR and CD46 are not used simultaneously. The exception is group D, in which adenoviruses use both receptors. After interaction with host cell receptors, there is integrin-mediated endocytosis [5]. The virus particles then undergo uncoating, with dissolution of the viral protein capsid in the endosome. This uncoating is also facilitated by the viral proteases, by the removal of various scaffolding proteins, a step crucial to virulence. This step renders a particular virion mature, as opposed to the immature form, which is more compact and stable and hence lacks infectivity given its inability to uncoat.

Subsequently, the adenovirus undergoes dynein-dependent translocation along microtubules through the cytoplasm [6] towards the nuclear pore complex with translocation into the nucleus [7]. Various components of both innate and acquired immunity block receptor interactions, virus entry, endosomal penetration and translocation. Specifically garnering interest is an arm of innate immunity, termed the human alpha defensins, which are found to directly bind to and inhibit the viral capsid, but further studies are needed to maximize the understanding and potential benefits of targeted immunotherapy [8].

## 4. Transmission

Transmission of adenovirus can occur by aerosol droplets, fecal–oral transmission, and contaminated fomites. Rarely seen is transmission via exposure to cervical canal secretions [9] during birth and in solid organ transplants [10], especially the liver and kidney. The virus can survive for extended periods on environmental surfaces and is resistant to lipid disinfectants because it is nonenveloped, however, it is inactivated by heat, formaldehyde, and bleach.

## 5. Epidemiology and Global Trends

In 1953, adenoviruses were first isolated by Rowe and colleagues while studying the growth of polioviruses in adenoidal tissue [11]. Adenovirus is found worldwide and infections can occur during any season. Young children, close-quartered populations such as crowded communities, schools, and military training camps [12], along with immunocompromised individuals are susceptible populations.

In the United States, disease surveillance is done by the National Adenovirus Type Reporting System (NATRS), a passive, laboratory-based surveillance system that coordinates the reporting of laboratory detections of human adenovirus types. Participating laboratories are encouraged to report typed human adenovirus detections quarterly to the Centers for Disease Control and Prevention (CDC) accompanied by limited demographic, clinical, and laboratory data. According to the most recent data, NATRS received reports for 2138 human adenoviral detections among specimens collected during 2003–2016 [13]. The data within the period 2003 to 2013 represented retrospective collection from reporting laboratories with formal surveillance starting in 2014. The most commonly reported specimen types were respiratory followed by ocular swabs [13]. The number of identified infections ranged from two in 2003 to 269 in 2014 when data collection formally began and increased to 362 in 2016. The report, however, was subject to limitations given that it was a passive system relying on voluntary participation from laboratories, possible biased data from outbreak investigations and nonrandom sampling, and limited laboratory capacities to test for specific adenoviral types and report in a timely fashion. Furthermore, given the self-limited disease course, patients often do not seek medical attention for the common cold and viral upper respiratory infections. If seen by a medical provider, a viral culture is often not warranted. The NATRS data is therefore likely to be an underrepresentation of the actual number of adenoviral cases. Therefore, the NATRS is currently focusing on increasing the number of participating laboratories, helping in building and maintaining capacity for virus identification and stressing timely reporting of data.

According to the WHO, the exact prevalence and incidence of adenoviral infections are unknown given that most of the cases are self-limited, seen by general practitioners and optometrists [14]. However, in the last few years, especially in Asia, a large-scale population, with outbreak numbers in the hundreds [15,16,17], has suffered from adenoviral infections presenting as acute respiratory distress syndrome (ARDS) in high frequency with outbreaks in Malaysia [16], South Korea [17] and China [15,18] among others. Immunocompromised patients have a greater risk of disseminated disease and increased mortality compared to patients with intact immune systems [19]. These outbreaks can be viewed as an opportunity for better study of the strains, predisposing factors, and the molecular biology [20] of the host–viral interactions responsible for disease and severity.

The diverse worldwide military [12] population remains a vulnerable target for the virus, as described by studies done on military personnel at various locations, from California to South Korea [21]. Interestingly, the immunocompetent nonmilitary, such as those in healthcare, have not been studied in detail, but existing research does not yet support routine vaccination [22]. In the United States from the years 1971 to 1999, military recruits were given a vaccine against adenovirus types 4 and 7 recognizing the health risks that these more common strains posed, but this was stopped along with production altogether when questions were raised about the efficacy and safety of vaccine. This led to a dramatic rise in number of infections noted in the military, with spikes in both febrile respiratory illnesses and number of hospitalizations due to adenovirus [23]. Subsequently, a surge of research gave way to reinvigorated use of a potential new vaccine, against the same strains. This live, oral vaccine was approved by the U.S. Food and Drug Administration in 2011, and thereafter the U.S. Department of Defense officially implemented its use, targeted specifically for military personnel aged 17 to 50 years [23,24]. As per a study done in 2014, the reintroduction of the vaccines resulted in a 100-fold decline in adenoviral disease burden in the first two years, from 5.8 to 0.02 cases per 1000 person-weeks, with a *p* value of <0.0001 [23].

## 6. Disease Spectrum

The spectrum of clinical disease differs with age, immune status and population characteristics. Upper respiratory tract infections (URI) are among the most common, while pneumonia and diarrhea are the most deadly in infants. Older children also present with URI most commonly, while pharyngoconjunctivitis (pink eye, swimming pool conjunctivitis which is non-purulent), hemorrhagic cystitis, and mesenteric adenitis are often seen as well. Epidemic keratoconjunctivitis (shipyard conjunctivitis) and ARDS are the main concerns in young military recruits. Immunocompromised patients are susceptible to the disease manifestations listed above, along with meningoencephalitis, interstitial nephritis, and gastroenteritis. During the last global survey, approximately one-fifth of all adenoviral infections reported to the World Health Organization (WHO) were attributed to serotypes 7 and 14, including outbreaks of severe disease [25].

Epidemic keratoconjunctivitis is one of the most frequent ocular manifestations of adenoviral infection, exhibiting ubiquitous distribution across the world. Due to its high frequency and the fact that many of the cases do not obtain medical attention, it is difficult to acquire precise statistical data on its incidence. Conjunctivitis is commonly caused by serotypes 8, 19 and 37 [26]. Patients usually complain of a prodrome of flu-like symptoms including fever, malaise, respiratory symptoms, diarrhea, and myalgia, sometimes with a recent history of ophthalmological examination or exposure within the family or at work. Ocular symptoms manifest with irritation, soreness, red eye, photophobia, foreign body sensation, excessive tearing, and in more severe cases, with ocular and periorbital pain and decreased visual acuity [27]. Ipsilateral pre-auricular lymphadenopathy is a classic finding as well. Bacterial superinfection, although not common, is particularly severe in pediatric patients and could lead to amblyopia [28].

Pharyngoconjunctival fever is primarily attributed to serotype 3 [29], which has caused outbreaks in children in schools and summer camps. The clinical features typically begin with a prodrome of fever, together with upper respiratory symptoms including pharyngitis, rhinitis, lymphadenopathy of the cervical group and also conjunctivitis with or without follicular reaction. It is usually self-limited, however, bacterial superinfection can be seen. Contaminated waters of swimming pools and reservoirs are the main sources of infection [29].

Adenoviral pneumonia is another clinically important entity, and serotypes 3, 7, 14, 21 and 55 have been associated with severe and complicated presentations [25,30]. Patients typically have a prodrome of fever, malaise, and myalgia, with a progressively worsening cough and dyspnea, unresponsive to empiric antibiotic therapy. Adenovirus was more commonly the causative agent of viral pneumonia in infants compared to older children and was also found to cause severe disease more often, with lethargy, diarrhea and vomiting, according to a study done on community-acquired pneumonia requiring hospitalization in the United States [31,32].

Extrapulmonary complications can occur, including meningoencephalitis, hepatitis, myocarditis, nephritis, neutropenia, and disseminated intravascular coagulation [32,33]. Increased activation of the immune system, especially macrophages, can result in hemophagocytic lymphohistiocytosis [34]. Severe pneumonia requiring mechanical ventilation and death is most often described in newborn, elderly or immunocompromised patients, but has also been reported in immunocompetent adults [30,35]. These present with severe symptoms, radiographic findings of bilateral pulmonary infiltrates similar to those of other viral pneumonias, negative bacterial cultures and non-response to antibiotic therapy.

Adenoviral pneumonia in immunocompetent individuals presents with chest Computerized Tomographic (CT) scan findings of subpleural and peribronchovascular consolidation with or without surrounding ground glass opacities [36,37]. A single center study done in a tertiary care hospital in Korea in 2016 suggested that large parenchymal involvement and presence of pleural effusion on CT are factors suggestive of progression to ARDS [38]. Some of the most common pathologic changes seen in the lungs were necrotizing bronchitis and bronchiolitis. The surviving bronchial epithelium frequently exhibited viral-induced proliferation, mononuclear cell infiltration, and hyaline membranes [39,40].

Severe cases of respiratory failure due to adenoviral pneumonia have been described in the literature, in which conventional mechanical ventilation had failed and patients required extracorporeal membrane oxygenation (ECMO) support [41]. A prospective study done in China in 2013–2014 described patients with persistent high fever, dyspnea and rapid progression to respiratory failure within two weeks, together with bilateral consolidations and infiltrates. These patients were found to have severe ARDS induced by adenovirus serotype 55. The study suggested viral load monitoring as a potential predictor of disease severity and the prompt use of ECMO for respiratory support if positive pressure ventilation failed [42].

Enteric viruses are major etiologic agents of acute gastroenteritis (AGE) among infants and young children worldwide. Enteric adenoviruses of subgroup F [43] and serotype 40 and 41 are recognized as frequent causes of acute gastroenteritis [44]. Outbreaks of AGE often occur in clusters, such as schools, camps, and daycare centers. Previous studies in children were mainly focused on community-acquired infection, but more recently, healthcare-associated adenoviral AGE is being more closely studied [43,45,46,47]. Along with rotavirus and norovirus, adenovirus has emerged as one of the leading causes of viral AGE in both these settings, with immunocompromised patients possibly infected with multiple viruses and shedding viruses in their stools for prolonged periods [46,47]. A study done in Austria from 2008 to 2013 with the aim of shedding light on the role of latent infections and intestinal reservoirs, evaluated the gastrointestinal (GI) tract of children with endoscopy, biopsy or stool samples, and identified 31% of children with persistence of infection, with the highest prevalence in the terminal ileum [48]. Intestinal lymphocytes, mainly in the lamina propria, were thought to be the nidus for persistence, while the epithelium was thought of as the main site for proliferation. Some studies suggest adenovirus as the main cause of hospital-acquired AGE [43].

In the urinary tract, adenovirus has been well known to cause acute hemorrhagic cystitis in children. Urogenital disease is less common in adults, but may present with urethritis. Hemorrhagic cystitis in children can be alarming at presentation, but is usually self-limiting and unaccompanied by the systemic signs of fever, hypertension, proteinuria associated with glomerulonephritis. The strains most often implicated in hemorrhagic cystitis are subgroup B serotypes 11 and 21 [49,50]. The duration of illness varies from four to five days to two weeks [49] with active viral shedding. In immunocompromised patients, serotypes 11, 34, and 35 have been found to cause tubulointerstitial nephritis [51] in addition to hemorrhagic cystitis.

Other adenoviral infections include meningitis [52,53], which presents rarely in immunocompetent adults and myocarditis [54,55], which is found more often in children.

In the immunocompromised [56] population, adenovirus is implicated in a wider spectrum of disease. Factors conferring a high risk of invasive infection and disseminated disease include stem cell or solid organ transplantation [57,58], congenital immunodeficiencies like severe combined immunodeficiency (SCID) syndrome [59] and acquired immunodeficiencies like human immunodeficiency virus (HIV) [60,61], which can predispose patients to more severe adenoviral disease. Patients undergoing chemotherapy [62], bone marrow transplantation, severe lymphopenia and those suffering from graft-versus-host disease are also predisposed to disseminated adenoviral infections. Interestingly, life-threatening disease currently appears to be relatively rare in acquired immunodeficiency associated with HIV infection, which is attributable to the advent of effective antiretroviral therapies, but the mortality rate of adenoviral infection can be as high as 55% in SCID [59,60,61]. In solid organ transplant recipients, adenoviral infections can range from asymptomatic to severe, with increased morbidity, graft loss and mortality [63]. Adenoviral infections can be acquired de novo or with the reactivation of latent virus from the recipient or the transplanted organ [64]. The symptoms of infection are variable but severe and even fatal courses have also been described [65]. However, invasive adenoviral infection usually does not correlate with organ rejection [64]. Adenoviral diseases are well characterized in hematopoietic stem cell transplant recipients. A wide range of clinical syndromes including pneumonia, colitis, hepatitis, hemorrhagic cystitis, tubulointerstitial nephritis, encephalitis, and disseminated disease have been frequently described. According to studies, allogenic stem cell transplantation seems to be a major risk factor for adenoviral infection, while infections in patients with autologous stem cell transplantation is rare [66].

## 7. Workup

In the past, adenovirus infections were difficult to diagnose and some infections were inappropriately treated with antibacterial agents. Direct fluorescent assay (DFA), as part of a viral respiratory panel, revolutionized prompt diagnosis of adenoviral infection [67]. After the development of DFA, viral cultures were less often utilized in clinical decision making [68]. The sample collection process is largely noninvasive, as a number of different samples including peripheral blood, stool, urine, bronchoalveolar fluid, nasopharyngeal aspirates or swabs can be readily used [52,54,59]. Low sensitivity was one of the major issues with DFA and viral cultures remained notoriously time consuming to impact clinical judgment and management. Thus, these methods are less commonly utilized in routine clinical screening in favor of PCR-based techniques, which are rapid and more reliable [69,70,71].

A prospective study of pediatric patients undergoing stem cell transplant revealed that detection of adenovirus at multiple sites can reflect the presence of invasive infection [69]. However, studies since have shown that peripheral blood is probably the most important source for the clinical surveillance of adenoviral infections in the immunocompromised setting [71,72]. A number of studies have attempted to correlate viral load to severity in order to determine when to start antiviral treatment, but further work is required in order to determine whether this approach will affect patient care [73,74]. Identification of adenoviruses by species, serotypes, and strains is relevant for epidemiological studies and for a wider rationale for the documentation of nosocomial outbreaks, but is often not indicated in clinical practice, given the self-limited disease course. Traditional serotyping methods have now been increasingly replaced by PCR techniques as well [75].

The gold standard for any infection, however, remains the viral culture [39,76]. All excepting a few viruses in the adenovirus family demonstrate a characteristic cytopathic effect in epithelial cell lines such as HeLa and HEp2. A few issues, however, make viral culture a tedious process. Samples can be readily taken from various sites, as mentioned above, but transportation is usually difficult as it should be done on special viral transport media, and on ice, to prevent the drying of samples, which may render them contaminated due to bacterial overgrowth. Asymptomatic shedding may also be an ongoing phenomenon, which complicates the differentiation of a carrier from an infected patient. Definitive diagnosis may sometimes require tissue biopsy to demonstrate characteristic histopathologic [77] evidence like intranuclear inclusions. Electron microscopy is occasionally used in the clinical setting, if most of the other tests are non-diagnostic, to directly demonstrate the virus structure. Rising antibody titers demonstrated in serological testing may also guide therapy in adenoviral infections.

In immunocompromised patients, the diagnosis is more challenging as patients can be asymptomatic carriers, have subclinical infection or have full-blown infection with disseminated features. Hence, clinical correlation becomes paramount along with confirmation of infection from more than one site, along with quantitative methods such as quantitative PCR and serological titers [78].

## 8. Management

Most adenovirus infections are self-limited. They rarely cause serious infection in adults and healthy children. However they can be life threatening to immunocompromised hosts, neonates and infants. Therefore most of the recommendations for treatment for these infections focus on immunocompromised patients, particularly allogenic transplant patients, who carry the greatest risk of life threatening infections.

## 9. Infection Control

The general recommendations that are made by the Center for Disease Control (CDC [79]) to the public for prevention of transmission include proper handwashing with soap and water, especially after contact with a person with any kind of flu-like illness, covering one’s mouth and nose when coughing or sneezing, avoiding touching one’s eyes, nose, or mouth with unwashed hands, avoiding close contact with people who are sick and staying home when people are sick themselves. Frequent handwashing is especially important in childcare settings and healthcare facilities. It is recommended to keep adequate levels of chlorine [80] in swimming pools to prevent outbreaks of conjunctivitis caused by adenoviruses.

## 10. Vaccination

Adenovirus vaccine is currently recommended for the U.S. military only and is not available to the general public. As mentioned above, vaccines against adenovirus types 4 and 7 was approved by the U.S. Food and Drug Administration in March 2011 [23,24]. These are live, oral, enteric-coated vaccines and are considered safe and effective in the prevention of epidemics of acute respiratory disease in military training camps. Two years into reintroduction and use, United States military trainees had a 100-fold decline in adenovirus disease burden [23].

## 11. Immunocompetent Patients with Mild Disease

There is no specific treatment for people with mild adenoviral infection. Most measures include supportive management of symptoms and complications. Anti-pyretics, analgesics, anti-emetics, adequate hydration, and oxygen supplementation as needed form the cornerstone of initial management.

## 12. Immunocompromised Patients and Antiviral Drugs

Antiviral therapy is generally reserved for patients with any form of immunocompromise, those receiving solid organ or stem cell transplants and those with disseminated or severe forms of the disease. In patients on immunosuppressive therapy due to solid organ transplants, there is an ongoing debate to preemptively [76] treat or to wait until the patient is clinically symptomatic [81]. This decision, however, changes to favor preemptive treatment in more cases than not when an allogenic stem cell transplant is carried out, until the immune system recovers back to the point of clearing any infection on its own [76,82].

## 13. Antiviral Agents

The most commonly used agent in today’s practice is cidofovir, which appears to be the most consistent active agent in vitro against adenovirus, more than other antiviral drugs, such as ganciclovir [83,84]. Similarly, most evidence for the in vivo efficacy of antiviral therapy against adenovirus in the preemptive setting is also available for cidofovir [85,86]. Cidofovir is a nucleotide analog of cytosine that selectively inhibits viral DNA polymerase and subsequently viral replication by competitive incorporation of its active metabolite cidofovir diphosphate into the viral DNA chain, disrupting synthesis and replication [87].

A mortality rate, as high as 75%, from invasive adenoviral disease was previously noted, following allogenic hematopoietic cell transplantation [88,89] prior to the use of cidofovir. Thereafter, cidofovir therapy has not only been associated with clinical improvement, but has also shown a survival benefit. The mortality rate from adenoviral disease was found to have dropped to less than 20 percent in severely immunocompromised patients with adenovirus infection treated with two or more doses of cidofovir [89].

Although encouraging outcomes are being seen with cidofovir use, low bioavailablity and nephrotoxicity remain limiting factors for widespread use. In a study done on pediatric patients undergoing lung transplants, the use of cidofovir in a modified dosing regimen along with Intra-Venous Immunoglobulin (IVIG) and renal protective measures showed no significant deterioration of renal function, highlighting a potentially safe and effective treatment regimen [90]. Experimental oral lipid–ester derivatives of cidofovir have also shown potential benefits while minimizing the nephrotoxicity potential in various studies. One such study conducted in 2003, which was carried out following concerns of the potential use of variola virus as a biological weapon and potentially devastating outbreaks of smallpox, mainly studied the effect of esterification of cidofovir with alkoxyalkanols and found that they increase the oral bioavailability and diminish the drug accumulation in the kidney, reducing nephrotoxicity [91].

Other agents such as Ganciclovir, ribavirin [76], vidarabine and foscarnet [92] have failed to show consistent activity against adenovirus in vitro for various reasons. Ganciclovir requires activation by phosphorylation to the monophosphate form by viral kinase, and since adenoviruses, in contrast to members of the herpesviridae, lack viral thymidine kinase, the efficacy of ganciclovir is modest in comparison to cidofovir [92].

In addition to antiviral therapy, there is ongoing research in T-cell-mediated immunotherapy [93]. The safety and efficacy of immunotherapy, along with the costs, have to be studied in much more detail before any recommendation is made on its clinical application. Pooled intravenous immune globulin (IVIG) has also been used as an adjunctive therapy in different immunocompromised settings and has shown some promising results [94]. More studies are required here as well.

## 14. Summary

The purpose of this review has been to present a concise outline of the biology, transmission, epidemiology, clinical presentation, diagnosis, prevention and therapy of one of the most important viruses seen in clinical practice today, the human adenovirus.

As the clinical presentations are nonspecific and vary over a wide spectrum of severity, a high index of suspicion based on the epidemiology and local prevalence is required by the clinician for diagnosis and either supportive or definitive therapy.

There are currently no recommendations on the screening of symptomatic immunocompetent patients by the US Preventative Services Task Force. There are, however, avenues where studies could be carried out for screening the immunocompromised, particularly high-risk, allogenic, stem cell transplant candidates, to identify chronic asymptomatic carriers.

Laboratory diagnosis is most commonly done by PCR, replacing traditional serotyping methods and cumbersome cultures.

Cidofovir remains the agent of choice in severe disseminated disease and derivatives are being explored as viable treatment options. The disease burden along with the potentially fatal outcomes seen, especially in immunocompromised persons, have paved the way for much research into various new avenues for pharmacotherapy and immunotherapy.
